# Understanding the Mechanism of Dysglycemia in a Fanconi-Bickel Syndrome Patient

**DOI:** 10.3389/fendo.2022.841788

**Published:** 2022-05-18

**Authors:** Sanaa Sharari, Mustapha Aouida, Idris Mohammed, Basma Haris, Ajaz Ahmad Bhat, Iman Hawari, Sabah Nisar, Igor Pavlovski, Kabir H. Biswas, Najeeb Syed, Selma Maacha, Jean-Charles Grivel, Maryam Saifaldeen, Johan Ericsson, Khalid Hussain

**Affiliations:** ^1^ Division of Biological and Biomedical Sciences, College of Health and Life Sciences, Hamad Bin Khalifa University (HBKU), Qatar Foundation, Doha, Qatar; ^2^ Department of Pediatric Medicine, Division of Endocrinology, Sidra Medicine, Doha, Qatar; ^3^ Department of Research, Sidra Medicine, Doha, Qatar; ^4^ School of Medicine and Medical Science, University College Dublin, Belfield, Ireland

**Keywords:** Fanconi-Bickel syndrome (FBS), dysglycemia, glucose transporter 2 (GLUT2), clustered regularly interspaced short palindromic repeats (CRISPR)- Cas9, sodium-glucose transport protein 2 (SGLT2)

## Abstract

Fanconi–Bickel Syndrome (FBS) is a rare disorder of carbohydrate metabolism that is characterized mainly by the accumulation of glycogen in the liver and kidney. It is inherited as an autosomal recessive disorder caused by mutations in the *SLC2A2* gene, which encodes for GLUT2. Patients with FBS have dysglycemia but the molecular mechanisms of dysglycemia are still not clearly understood. Therefore, we aimed to understand the underlying molecular mechanisms of dysglycemia in a patient with FBS. Genomic DNA was isolated from a peripheral blood sample and analyzed by whole genome and Sanger sequencing. CRISPR-Cas9 was used to introduce a mutation that mimics the patient’s mutation in a human kidney cell line expressing GLUT2 (HEK293T). Mutant cells were used for molecular analysis to investigate the effects of the mutation on the expression and function of GLUT2, as well as the expression of other genes implicated in dysglycemia. The patient was found to have a homozygous nonsense mutation (c.901C>T, R301X) in the *SLC2A2* gene. CRISPR-Cas9 successfully mimicked the patient’s mutation in HEK293T cells. The mutant cells showed overexpression of a dysfunctional GLUT2 protein, resulting in reduced glucose release activity and enhanced intracellular glucose accumulation. In addition, other glucose transporters (SGLT1 and SGLT2 in the kidney) were found to be induced in the mutant cells. These findings suggest the last loops (loops 9-12) of GLUT2 are essential for glucose transport activity and indicate that GLUT2 dysfunction is associated with dysglycemia in FBS.

## Introduction

The classical phenotype of Fanconi-Bickel Syndrome (FBS) was initially described by 1 (1). GLUT2 mutations were first described in three FBS patients, including the original patient in 1997 (2). More than 100 FBS cases with different *SLC2A2* mutations; nonsense, missense, Fs/InDel, intronic, and compound heterozygous variants have been reported so far (3–8). *SLC2A2* gene consists of 11 exons and 10 introns and encodes for the GLUT2 transmembrane protein (524 amino acids) (SLC2A2-201 ENST00000314251.8) (9). GLUT2 is a low affinity facilitated glucose transporter and expressed in tissues that have a role in glucose homeostasis. GLUT2 in human and rat liver is considered the primary transporter for glucose uptake and storage as glycogen during the feeding state, and to release glucose either by glycogenolysis or gluconeogenesis during the fasting state (10, 11). Glycogen storage, post-prandial hyperglycemia and fasting hypoglycemia in FBS patients can be explained due to a disturbance in glucose transport and metabolism in the liver. Moreover, GLUT2 in the kidney releases filtered glucose into the blood circulation. Previous studies suggested that GLUT2 dysfunction in the kidney is associated with glycogen storage and glycosuria, which are the main symptoms found in FBS patients (12, 13). Furthermore, GLUT2 is the major glucose transporter in the rat beta cells and is suggested to play a role in glucose uptake and insulin secretion. However, GLUT2 is expressed at low levels in human beta cells, and its role is not well studied and is still controversial (3, 14, 15). FBS patients develop dysglycemia (glucose intolerance, post-prandial hyperglycemia, fasting hypoglycemia, transient neonatal diabetes, frank diabetes mellitus, and gestational diabetes) with different severity regardless of the type of mutation. The molecular mechanisms of dysglycemia in FBS are not well understood (4). In this study, we aimed to mimic a patient’s GLUT2 mutation in human embryonic kidney cells (HEK293T) to investigates the role of GLUT2 in dysglycemia associated with FBS.

## Materials and Methods

### Patient Recruitment and Genetic Analysis

This study was approved by the Institutional Review Board for the Protection of Human Subjects, Sidra Medicine, Qatar and written informed consent was obtained for the study. Clinical information was collected and genomic DNA of the patient and parents was isolated from peripheral blood samples using QIAamp DNA Blood Maxi Kit (Qiagen). Whole-genome sequencing (WGS 30x) using the Illumina HiSeq platform was performed. Sanger sequencing was used to confirm the mutation in the patient and both parents using primers (**Table S1**). Snapgene software was used for Sanger sequencing analysis.

### CRISPR-Cas9

Clustered Regularly Interspaced Short Pallindromic Repeats (CRISPR)-Cas9 system was used for GLUT2 gene modification in human embryonic kidney cells (HEK293T). Different guide RNAs (gRNAs) close to the patient mutation identified by the PAM sequence (NGG) were designed (**Table S1**). The gRNA construct containing the 20 nucleotides target CRISPR sequence (crRNA) and the tracer sequence (tracrRNA) was generated. The genome-editing protocol described by Lee et al. (16) was used with the few changes to introduce GLUT2 edits in HEK293T cells. To form the gRNA, sense and antisense oligonucleotides with BbsI overhangs (**Table S1**) were phosphorylated with T4 polynucleotide kinase. The Cas9 plasmid (pX330-U6-Chimeric_BB-CBh-hSpCas9 was a gift from Feng Zhang (Addgene plasmid # 42230; http://n2t.net/addgene:42230; RRID : Addgene_42230)) was digested with BbsI and purified from a 1% agarose gel using a gel extraction kit (QIAEX II). Subsequently, ligation of the digested pX330-U6-Chimeric_BB-CBh-hSpCas9 plasmid with the annealed gRNAs were performed at 16°C overnight, using T4 DNA ligase (Invitrogen). The gRNA-Cas9 plasmid was transformed into chemically competent TOP10 bacteria (ThermoFisher), and screened on LB agar plates supplemented with 100 μg/mL ampicillin. Positive Cas9-gRNA plasmids were validated by Sanger sequencing using CRISPR_Seq primers (**Table S1**) following plasmids extraction using QIAprep Spin Miniprep Kit (QIAGEN). Further amplification of the positive plasmids was performed using Endofree Plasmid Maxi Kit (QIAGEN). To detect the most efficient gRNA, HEK293T cells were used. In short, HEK293T cells were transfected with the different gRNA-Cas9 plasmids using FuGENE^®^ HD Transfection Reagent (Promega). Eighty thousand cells were seeded in a 24-well plate, and 1ug of gRNA-Cas9 plasmid was transfected with 3.4 μl of FuGENE HD transfection reagent. Genomic DNA was extracted from the transfected HEK293T cells after 2-3 days using Genomic DNA QuickExtract (EpiCentre, Madison, WI, USA) and amplified by Amplitaq reaction at 55°C using genomic GLUT2 forward and reverse primers (**Table S1**), and purified following the manufacturer’s protocol (QIAquick PCR Purification Kit (50), QIAGEN)). T7 endonuclease assays were performed to detect heteroduplex DNA resulting from gene editing (one wild-type and one mutant DNA strand). 200 ng of purified DNA was denatured and annealed in a thermomixer (10 minutes at 95°C, followed by a gradual decrease in temperature to 25°C). Reannealed DNA was then mixed with an enzyme master mix containing 0.5 μL of T7 Endonuclease I (New England Biolabs), 0.2 μL of 10× NEBuffer #2, and 1.3 μL of sterile distilled water and incubated at 37°C for 60 minutes. The reaction was stopped immediately after incubation by the addition of 6 μL of an EDTA-containing stop solution. The entire reaction was loaded on a 2% agarose gel and stained with SyberSafe. The mutations introduced by the individual gRNAs were further analyzed by Sanger sequencing of bacterial colonies following TA cloning (TOPO^®^ TA Cloning^®^ Kit, Invitrogen) using M13 primers (**Table S1**). gRNA with the highest editing activity was used to transfect HEK293T cells for single cell originated clone isolation and expansion. Lastly, Sanger sequencing of several HEK293T cell colonies was performed to identify the specific gene edits generated. The mutation in the selected clone was confirmed in the first three passages to exclude any contamination from wild-type cells.

### Growth Assay

To visually monitor cell growth, 300,000 wild-type (WT)/mutant HEK293Tcells were seeded in 60 mm culture dishes, and images were taken by a 10X microscope on days 1 and 4. Edu Cell Proliferation Assay (EdU-647, Merck Millipore) was performed on day 2 using 60,000 WT/mutant cells in 4-well glass chambers slides. Edu (50 μM) was added to the test chambers and incubated for 3 hours. The cells were fixed with 4% PFA for 15 minutes and permeabilized with 0.5% Triton X-100 in PBS for 20 minutes. Then, 100 μL of reaction cocktail was added for 30 minutes and kept in the dark, followed by washing with 3% BSA in PBS and stained with DAPI. Fluorescence images of stained and fixed cells were acquired using a 60x oil objective in an Eclipse Ti inverted microscope (Nikon, Tokyo, Japan) fitted with a CSU-X1A confocal spinning disk unit (Yokogawa, Tokyo, Japan), a Visitron Systems (VS) – Laser Merge System Laser Combiner including VS-ViRTEx experiment control unit, and a pco.edge 4.2 scientific CMOS camera (PCO AG, Kelheim, Germany). Images were collected in the VisiView (Visitron Systems GmbH, Puchheim, Germany) and analyzed with Fiji/ImageJ (NIH, Bethesda, MD) (17, 18). Briefly, 16-bit confocal fluorescence images were converted into 8-bit images, and individual cell nuclei were manually selected using the freehand selection tool. Selected regions of interest (ROI) were added to the ROI manager, and background-subtracted mean intensities were calculated and plotted for each cell type.

### Flow Cytometry

We used 50,000 WT/mutant HEK293T cells to measure the expression of GLUT2. Cells were fixed with 4% PFA for 15 minutes and then permeabilized for 20 minutes. A small portion of each cell type was kept unstained, while the remaining cells were incubated with 10µl of anti-hGLUT2 PE-conjugated mouse IgG2a antibody (R&D SYSTEMS, FAB14148) or 5µl of PE mouse IgG1 control antibody (400112, BioLegend) and remaining volume of 100μl of Brilliant staining buffer (BD Biosciences) for 15 minutes in the dark. The stained cells were washed once with staining buffer (BioLegend), and the signal was measured on a BD Symphony A5 instrument.

### qRTPCR

The expression of other glucose transporters (SGLT1, SGLT2, and GLUT1) in WT and mutant HEK293T cells was examined quantitatively. RNA from WT/mutant HEK293Tcells was extracted following the manufacturer’s protocol (RNeasy Mini Kit, Qiagen) and normalized to 2 µg for cDNA synthesis. Three µL cDNA products were added to Fast 96-Well reaction plates (0.1 mL) (Applied Biosystems) and amplified using SYBR^R^ Green PCR master mix (Applied Biosystem) at primer-specific Tm (**Table S1**). The mRNA levels were quantified using a QuantStudio™ 12K Flex SystemBlock 96-well instrument.

### Glucose Uptake Assay

To test the glucose uptake activity, 500,000 WT/mutant cells were incubated in low glucose medium for 3 hours, followed by incubation with 1 mM 2-DG for 1 hour. Cells were lysed with extraction buffer at 85°C for 40 minutes. Next, the reaction mixtures were neutralized with 10 μL neutralizing buffer. Intracellular glucose levels were analyzed as recommended by the manufacturer (Glucose Uptake Assay Kit (Colorimetric, Abcam). The absorbance was measured at 412 nm using a microplate reader (Flaoster, Omega).

### Statistical Values

All results were assessed three times, and the average of three values is given as the Standard deviation. P-value was calculated using two-tailed t-test. P-value <0.05 is considered as a significant difference.

## Results

### Clinical Information and Genetic Testing Results

**Figure 1:**
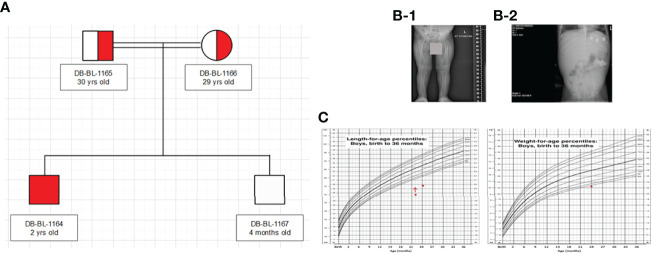
Clinical characteristics of patient: **(A)** Family Pedigree **(B)** Radiological findings (x-ray showed rickets (1), and hepatomegaly (2). **(C)** Growth charts (According to CDC length chart, patient has short stature (dot) responding to growth hormone (red arrow), and underweight (dot).

**Figure 2:**
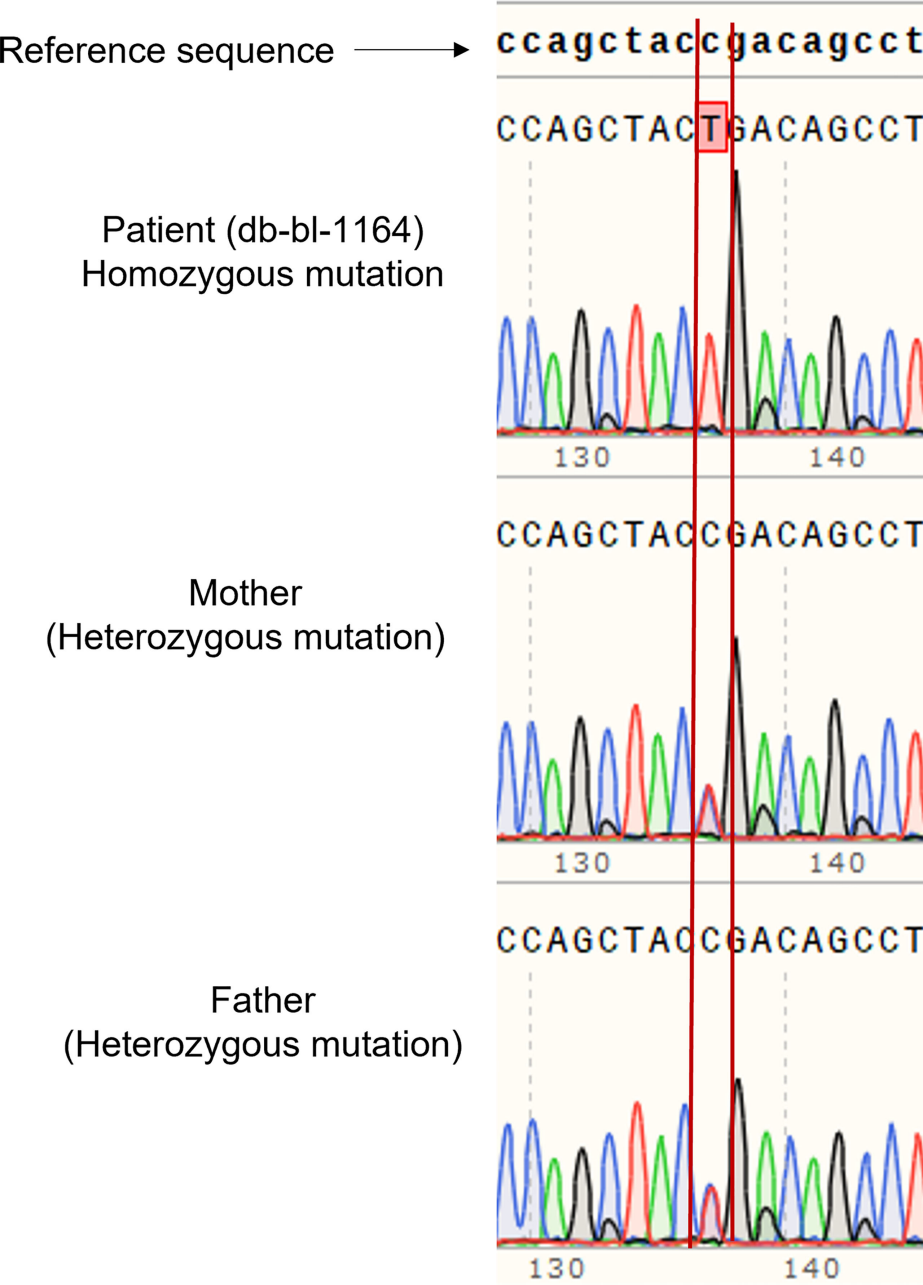
Genetic analysis of patient and parents. Sanger sequencing of DNA of the patient showed homozygous mutation of *SLC2A2* (c.901C>T, R301X), and the parents are carriers.

We report a 2-year-old Palestinian boy born to consanguineous parents with FBS (**Figure 1A**). He was born full term by normal vaginal delivery with a birth weight of 2.8 Kg (3^rd^ centile) and length 49 cm (15^th^ centile). Maternal history was significant for gestational diabetes mellitus. His newborn screening showed high galactose levels with normal GALT activity. He was followed up by a metabolic team and started on a special formula feed since birth. On day 18 after birth, the patient presented with poor feeding, vomiting, and polyuria. His biochemical tests showed severe metabolic acidosis with electrolyte imbalance, glycosuria, proteinuria, and phosphaturia (**Table 1**). In addition, the patient displayed dysglycemia (fasting hypoglycemia and post-prandial hyperglycemia, with low levels of C-peptide and insulin). HbA1c levels were high, and diabetes mellitus Type 1 evaluation was negative for all autoantibodies (**Table 1**). At the age of 5 months, the patient was found to have hepatomegaly (**Figure 1B-2**) and impaired liver function tests (**Table 1**) with liver biopsy showing stage 1 fibrosis. The lipid profile was normal except for elevated triglyceride levels (**Table 1**). In addition, the patient had failure to thrive and also developed rickets (**Figure 1B-1**). An MRI of the brain showed features suggestive of anterior pituitary (adenohypophysis) hypoplasia. As for the full pituitary hormonal tests, they were normal except for low IGF-1 levels (**Table 1**) and the patient was diagnosed with growth hormone (GH) deficiency following a GH provocation test (data not shown). Recombinant GH therapy was started for the patient and monitored over six months, where he showed an increase in height to 16cm/year compared to 3 cm/year before therapy (**Figure 1C**). In addition, the patient received multiple medications for electrolyte imbalance (sodium bicarbonate, potassium, phosphorous, and vitamin D). The patient’s whole genome sequencing revealed a homozygous mutation (c.901C>T, R301X) (NM_000340) in the *SLC2A2* gene, with both parents being carriers of the same mutation. The mutation was confirmed by Sanger sequencing of patients DNA (**Figure 2**). **Figure S1** illustrates the expected truncated GLUT2 topology for the patient.

**Table 1:** Summary of biochemical tests for the patient.

Investigation	Test value	Normal range
**Electrolyte levels and Urine analysis**		
**Serum phosphorus (mmol/L)**	0.80	0.93-1.64
**Serum calcium (mmol/L)**	1.7	2.2 -2.7
**Serum Sodium (mmol/L)**	132	134-146
**Serum Potassium (mmol/L)**	3.1	3.5-5.0
**BUN (mmol/L)**	2.6	1.2-6.3
**Creatinine (µmol/l)**	25	35-58
**Urinalysis**	Proteinuria (+2) glycosuria (+3)	
	Phosphaturia	
**Liver function tests**		
**Alanine amino transferase (ALT) (IU/L)**	82	8-22
**Aspartate transaminase (AST) (IU/L)**	110	0-30
**Alkaline phosphatase (IU/L)**	410	48-95
**Blood glucose tests**		
**Fasting glucose (mmol/l)**	2.1	3.5-5.5
**2 hours post OGTT (mmol/l)**	20	7.8-11.1
**C-Peptide (ng/ml) [At Diagnosis]**	0.33	0.78-5.19
**Insulin (pmol/l) [At Diagnosis]**	6	111-1153
**HbA1c% [At Diagnosis]**	8.1	4.8-6.0
**Diabetes mellitus Type 1 evaluation (GAD65, Insulin, IA-2, ZnT8 Abs test)**	Negative	–
**Miscellaneous Hormone Profile**		
**TSH (mIU/L)**	3.34	0.4-4.0
**PTH intact (pmol/l)**	1.8	2.0- 6.8
**IGF-1 (mcg/dl)**	<3.0	27.4-113.5
**Lipid profile**		
**Cholesterol (mmol/l)**	3.5	<5.18
**Triglyceride (mmol/l)**	3.2	<1.7
**High Density Lipoprotein (HDL-C) (mmol/l)**	0.7	>1.17
**Low Density Lipoprotein LDL (mmol/l)**	2	<2.6

### CRISPR-Cas9 Technology to Mimic GLUT2 Patient’s Mutation in HEK293T Cells

**Figure 3:**
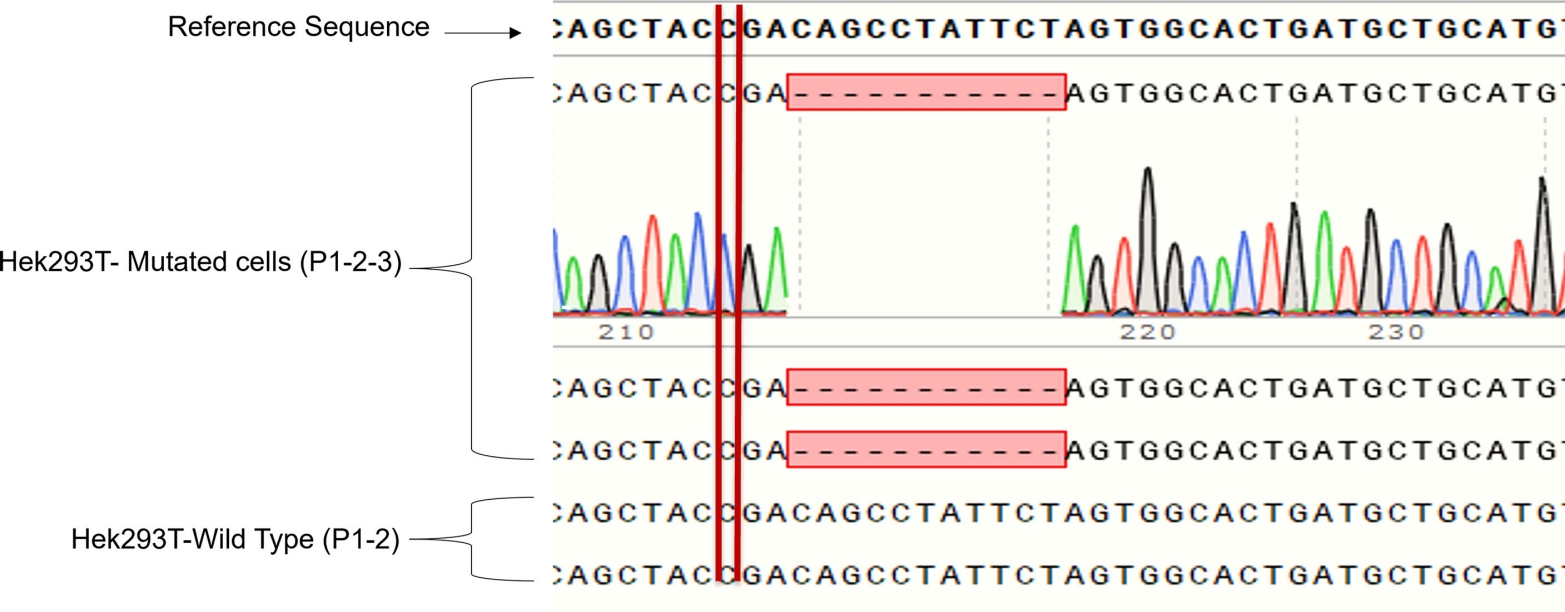
Sanger sequencing of the first three passages of sorted HEK293T cells with GLUT2 mutation. The deletion mutation was confirmed in the first three passages of the sorted cell colony to validate cell genotype.

We cloned four different gRNAs into a Cas9-expressing plasmid to edit GLUT2 in HEK293T cells (**Figure S2**). gRNA3 was found to be the most efficient to edit GLUT2 (**Figure S3**) with different insertion and deletion mutations in GLUT2 (**Figure S4**). The selected HEK293T colony displayed a deletion mutation of 11 nucleotides, located two nucleotides after the patient mutation site (c. 901 C>T, R301X) (**Figure 3**). This resulted in a frameshift in GLUT2 and introduced a stop codon at c. 1164. Both the mutant and the WT cell lines were monitored by Sanger sequencing for few passages, all of which revealed a clean and identical sequences (**Figure 3**). Mutant cell line showed only the recurrent deletion trace; no WT nor other traces was detected, and therefore the cell line was characterized with a homozygous mutation in GLUT2 close to the mutation site identified in the patient. These cells were subsequently used to evaluate the impact of GLUT2 mutation on glucose transport activity.

### Proliferation Rate of WT and Mutant Cells

**Figure 4:**
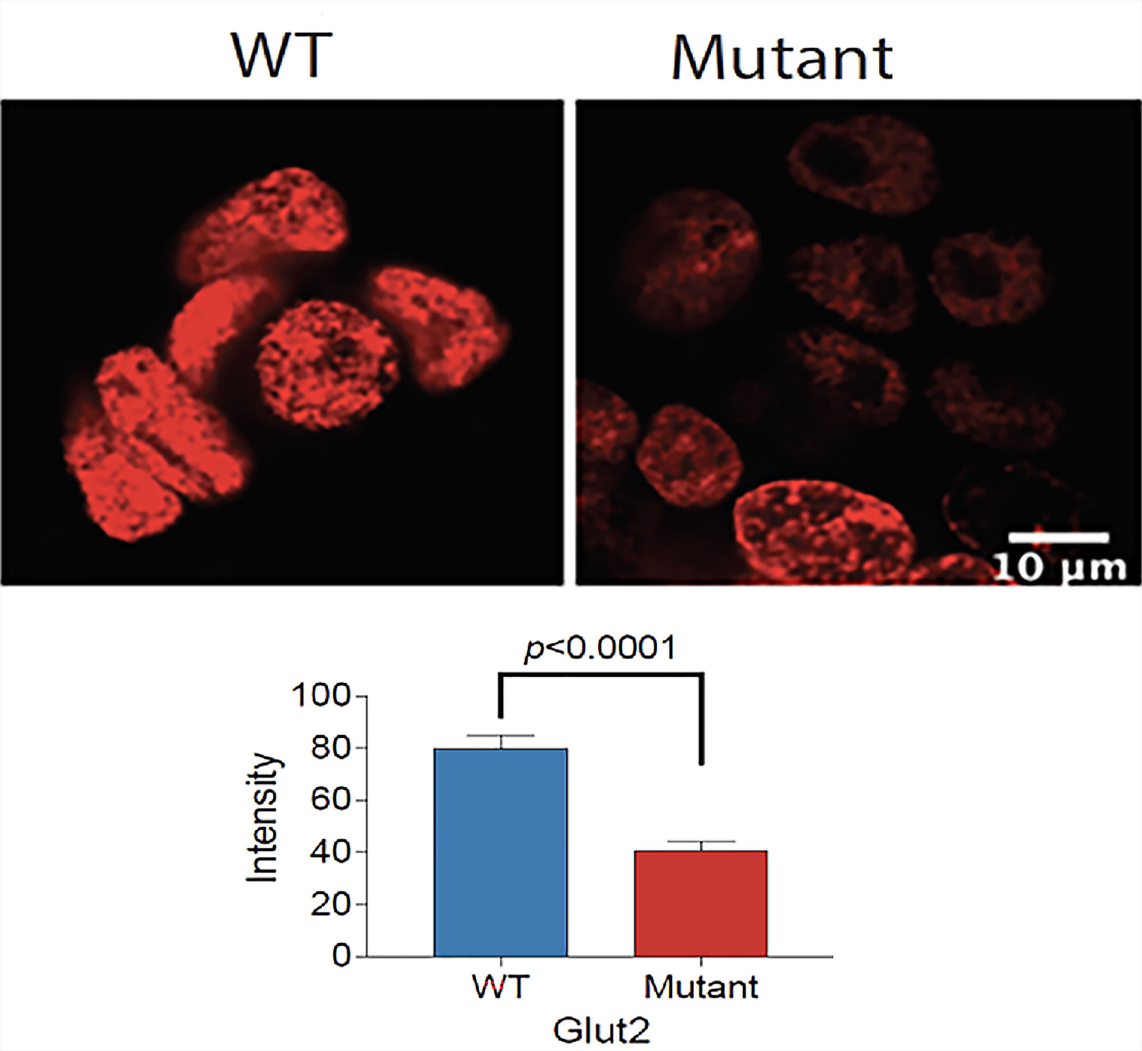
Reduced proliferation of GLUT2 mutant cells. Top panel: Confocal fluorescence images of WT and mutant GLUT2 expressing HEK293T cell nuclei showing reduced Edu incorporation. Lower panel: Graph showing Edu intensity in the nuclei of WT and mutant GLUT2 expressing HEK293T showing significantly lower Edu intensities (p<0.0001). Analysis based on single cell nuclei measurement.

We cultured the same number of WT and mutant HEK293T cells in complete DMEM medium supplemented with 10% fetal bovine serum and 1% P/S to assess the difference in growth. Both cell lines displayed similar morphology; however, the GLUT2 mutant cells grew slower than the WT cells at day 1 and 4 (**Figure S5**), suggesting that the mutation of GLUT2 affected cell growth. To further explore this possibility, we performed Edu incorporation assays to investigate the difference in proliferation rate between WT and mutant cells. The Edu signal (red fluorescence) was strong in wild-type cells when compared to mutant cells (**Figure 4**, top panel). Quantification of the intensity of the Edu signal suggested that wild-type cells proliferated at a significantly faster rate than the mutant cells (**Figure 4**, lower panel).

### GLUT2 Expression in Wild-Type and Mutant HEK293T

**Figure 5:**
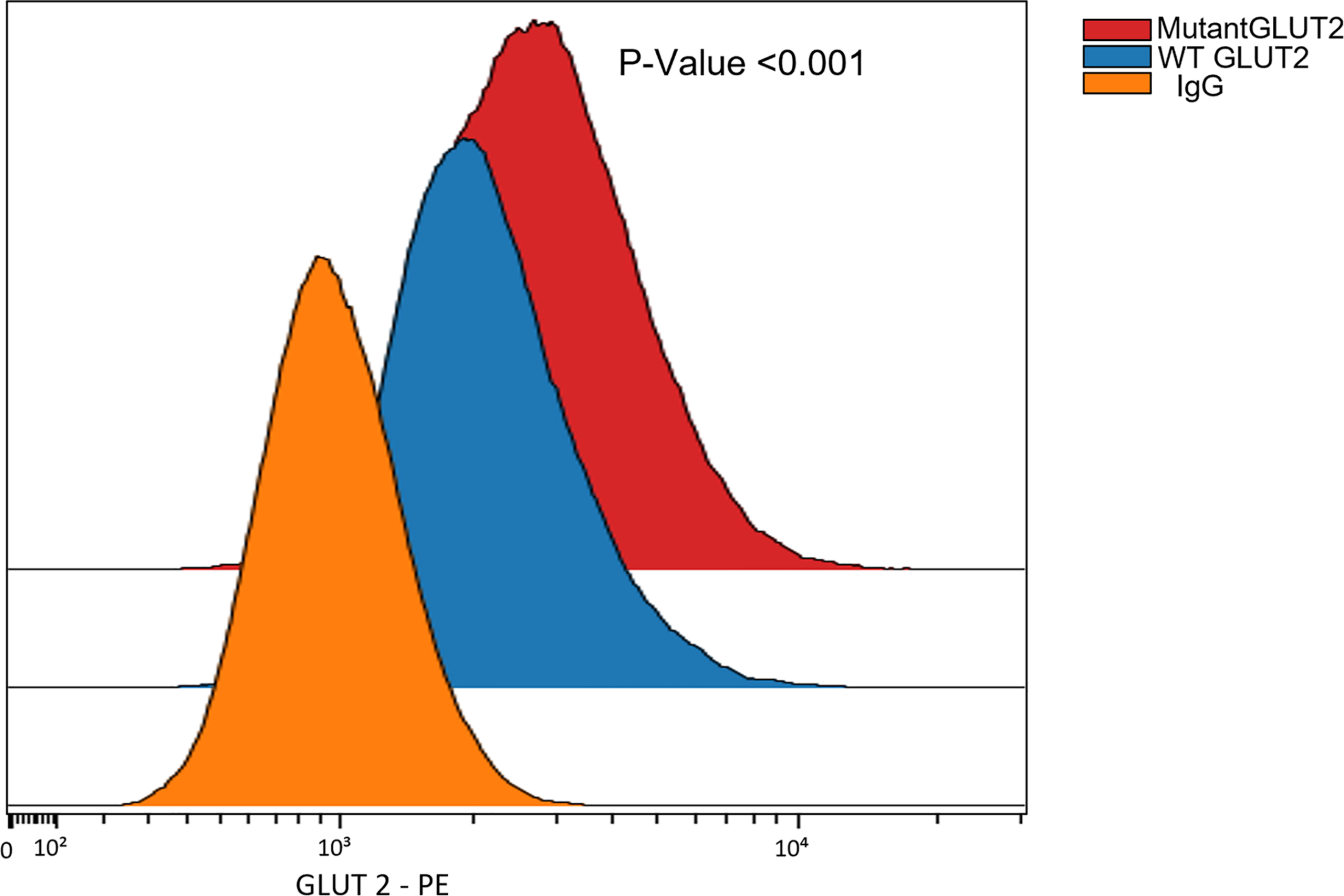
Flow cytometry to assess the expression of GLUT2 protein in WT and mutant HEK293T cells. The expression of GLUT2 was significantly increased in the mutant cells. P-Value **<**0.001 using nonparametric Mann-Whitney test.

The expression of GLUT2 protein in wild-type and mutant cells was monitored using flow cytometry. Surprisingly, the expression of GLUT2 was significantly increased in the mutant cells in comparison to wild-type cells (**Figure 5**).

### The Expression of Other Glucose Transporters in HEK293T Cells

**Figure 6:**
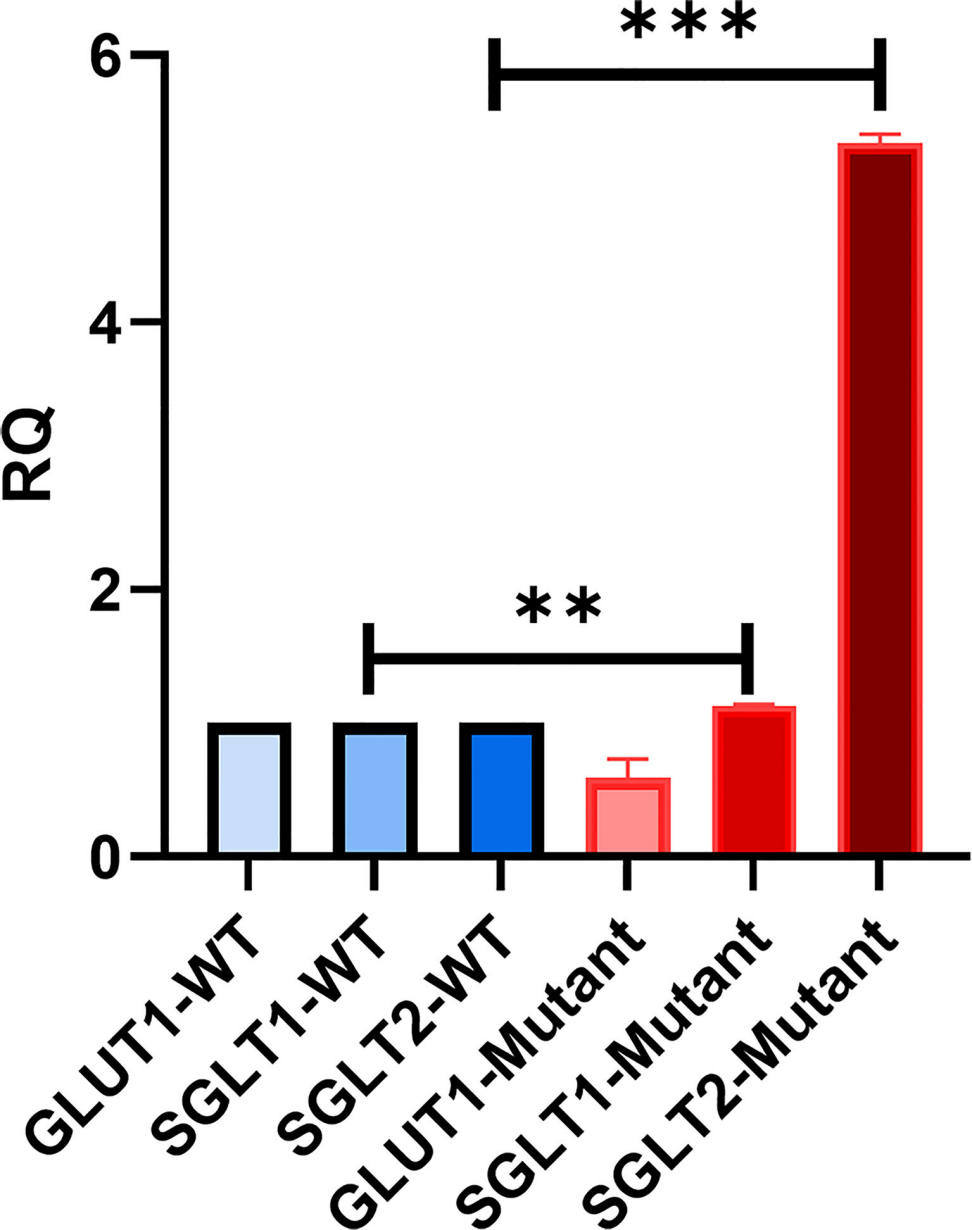
qRT-PCR to assess the expression of other glucose transporters in HEK293T. GLUT2 mutation stimulates the expression of SGLT2. Significant induction of SGLT1 and SGLT2 in mutant cells in comparison to WT cells. Down-regulation of GLUT1 expression in mutant cells in comparison to WT cells. P-value was calculated using two-tailed t-test and presented with a ‘‘*’’ in the graph. **P values less than 0.01; ***P values less than 0.001.

We were interested in investigating the expression of other glucose transporters in the GLUT2 mutant cells. Therefore, the gene expression of SGLT1, GLUT1, and SGLT2 was assessed using qRT-PCR. We amplified an equal amount of cDNA from normalized high-quality RNA (2μg) extracted from WT and mutant HEK293T cells. The mutant cells displayed a slight increase in the expression of SGLT1 (**Figure 6**). We were unable to detect any expression of SGLT2 in WT cells, while the same transporter was expressed at relatively higher levels in the mutant cells (**Figure 6**). The expression of GLUT1 was slightly reduced in the mutant cells compared to WT cells (**Figure 6**). Thus, our results suggest that mutation of the *SLC2A2* gene (GLUT2) in HEK293T cells results in the overexpression of a dysfunctional GLUT2 protein and enhanced expression of SGLT2, which could result in increased accumulation of intracellular glucose.

### Glucose Uptake in WT and Mutant HEK293T Cells

**Figure 7:**
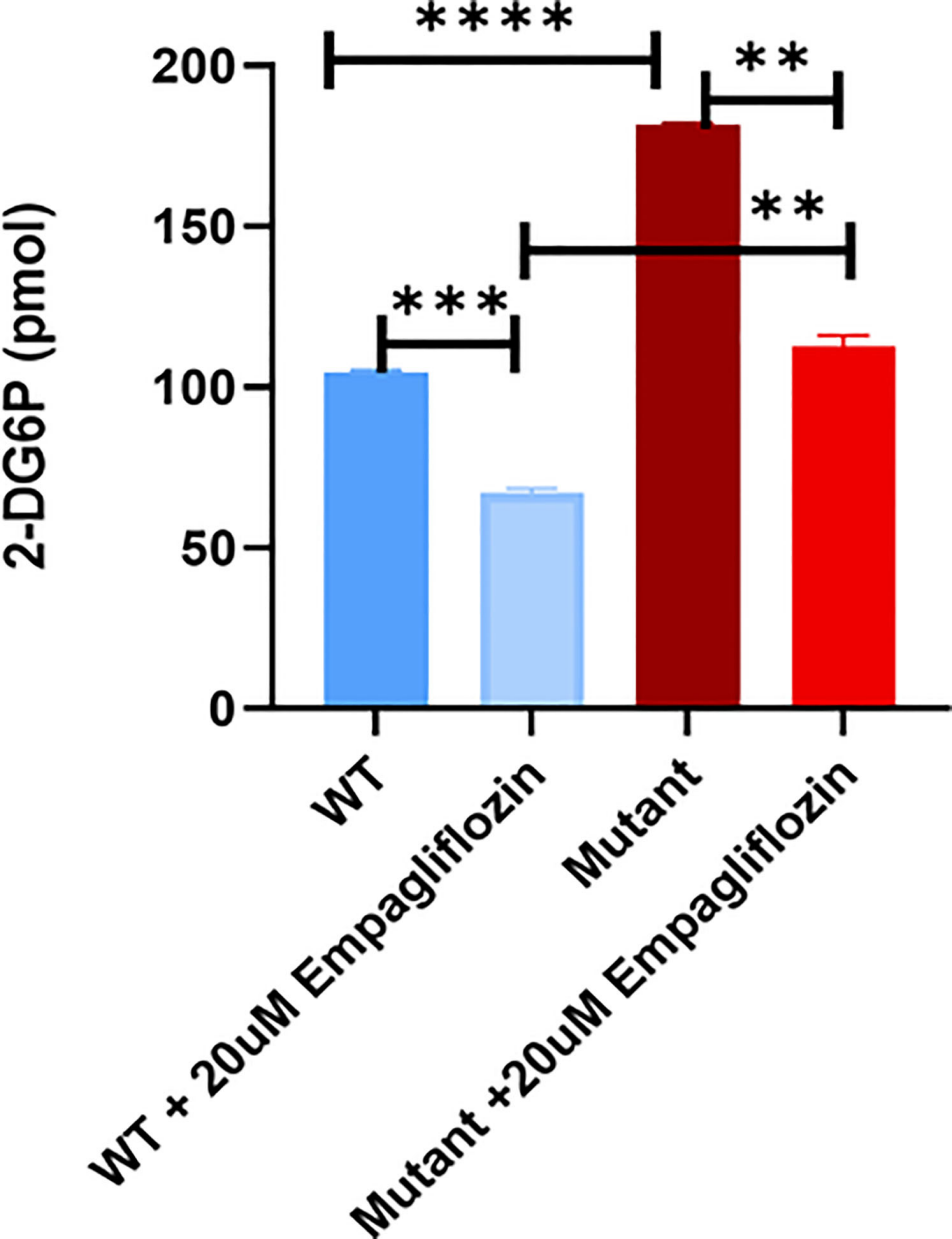
Glucose uptake test in WT and mutant HEK293T cells using 2-DG. Mutant cells have significantly increased glucose accumulation in comparison to WT. Treatment of the cells with the SGLT2 inhibitor (Empagliflozin) confirmed the GLUT2 release activity in the mutant cells is disturbed. P-value was calculated using two-tailed t-test and presented with a ‘‘*’’ in the graph. **P values less than 0.01; ***P values less than 0.001; ****P values less than 0.0001.

To study the effect of the GLUT2 mutation on glucose uptake, cells were incubated with 1 mM 2-DG. Interestingly, the intracellular levels of 2-DG were significantly higher in the mutant cells compared to WT cells (**Figure 7**). To determine whether the increased accumulation of glucose in the mutant cells was due to an increase in SGLT2 expression (**Figure 6**) or due to the inhibition of GLUT2-mediated glucose export, the glucose uptake assay was repeated in the presence of the SGLT2 inhibitor empagliflozin. As illustrated in **Figure 7**, the SGLT2 inhibitor reduced glucose accumulation in both cell lines. Importantly, the accumulation of intracellular glucose remained higher in the mutant cells even in the presence of the inhibitor, suggesting that the mutant GLUT2 protein is less efficient in glucose export. Thus, we suggest that the last four transmembrane domains (domains 9-12) of GLUT2 are essential in glucose export activity in kidney cells, and treatment with SGLT2 inhibitors could attenuate the dysglycemia observed in FBS patients.

## Discussion

Dysglycemia is observed in virtually all patients with FBS at some stage of their lives. The pattern of dysglycemia in FBS patients can range from fasting hypoglycemia, post-prandial hyperglycemia, and diabetes mellitus. However, the underlying biochemical and molecular mechanisms of dysglycemia are not well understood. This study aimed to understand how disturbances in GLUT2 function is associated with dysglycemia in an FBS patient. For the first time, we use CRISPR-Cas9 genome editing to mimic the mutation identified in a FBS patient in HEK293T cells, a human embryonic kidney cell line. In the kidney, the main role of GLUT2 is to transport glucose back to the circulation, thereby preventing glucose loss. Using this novel FBS model, we demonstrate that the gene edited cells accumulate more glucose than the wild-type cells, likely as a result of reduced glucose export. Our results could therefore explain, at least in part, the accumulation of glycogen in the kidneys in some FBS patients.

A recent study aimed at understanding the molecular mechanisms of dysglycemia in FBS used HEK293 cells transfected with constructs expressing either wild-type or FBS-associated mutant GLUT2. All mutants displayed the same fructose uptake activity as the WT protein, except for p.Thr198Lys, which displayed a small decrease in uptake activity (19). Although informative, this work was based on the overexpression of mutant proteins on top of wild-type endogenous GLUT2 expressed in HEK293. In contrast, the current study aimed to understand the molecular mechanisms of dysglycemia in FBS patients by mimicking the GLUT2 mutation we recently identified in a patient. To achieve this aim, we used CRISPR-Cas9 gene editing to introduce a mutation in *SLC2A2* in HEK293T cells. One of our gRNAs introduced a deletion mutation close to the mutation found in our patient, thereby causing a frameshift and a stop codon at nucleotide 1164 (**Figure 3**). The mutant cells displayed slower proliferation than the wild-type cells, which suggest that GLUT2 could affect cell growth (**Figure 4**). This is an interesting observation as most patients with FBS have short stature and failure to thrive and thus needs further investigations.

Proximal tubular cells augment glucotoxicity during hyperglycemia, either by increased glucose reabsorption or intracellular glucose accumulation (20). Song et al. suggested that the inhibition of SGLT1 results in mild glycosuria that is enhanced in response to SGLT2 inhibition (21). Chhabra et al. reported glycosuria in mice with hypothalamic melanocortin deficiency due to decreased GLUT2 expression (22). In addition, Hinden et al. showed that the inhibition of the cannabinoid-1 receptor (CB1R) leads to down-regulation of GLUT2. Hence, the translocation of GLUT2 to the apical membrane of renal proximal tubular cells (RPTCs) was affected, causing a decrease in glucose reabsorption and glycosuria in diabetic mice (23). Moreover, de Souza Cordeiro et al. specifically knocked out GLUT2 in mice and reported that GLUT2 dysfunction was associated with glycosuria and improved glucose tolerance (24). Another study showed an increase in glucose uptake by the apical movement of GLUT2 in rats treated with streptozotocin to induce diabetes; this effect disappeared in response to overnight fasting (25). In addition, a separate study demonstrated that the expression and activity of SGLT2 and GLUT2 were enhanced in human exfoliated proximal tubular epithelial cells (HEPTECs) isolated from patients with type 2 diabetes mellitus in comparison to healthy controls (26). Recently, Jiang et al. explained the potential role of renal SGLT2 and GLUT2 in the pathology of gestational diabetes mellitus (GDM) in a mouse model exposed to a high-fat diet (27).

During euglycemia, glucose reabsorption in the kidneys occurs primarily *via* SGLT2 and secondarily through SGLT1 (28). GLUT2 and GLUT1 release glucose across the basolateral membrane into the bloodstream. Glycosuria occurs once the glucose levels in the blood exceed the maximum re-absorptive capability of the kidneys. During hyperglycemia, SGLT2 and SGLT1 activities are enhanced due to increased glucose glomerular filtration. Moreover, protein kinase C stimulates the translocation of GLUT2 to the brush border membrane, which may increase glucose reabsorption (28). Wu et al. concluded that the last four transmembrane domains (domains 9 to 12) play a major role in glucose transport activity using Xenopus oocytes and mammalian cells (29). The latter conclusion is supported by the data reported in the current study. The GLUT2 mutant generated in our study contains a premature stop codon upstream of transmembrane domains 9-12 and the mutant protein displays reduced glucose transport activity (**Figure 7**).

In our study, mutation of GLUT2 in HEK293T cells resulted in the upregulation of GLUT2 protein (**Figure 5**). This was a rather surprising observation, since the gene editing introduced a premature stop codon in GLUT2. Thus, the exact nature of the GLUT2 protein in the edited cells will require further studies.

We found that the mRNA levels of SGLT2 were very low in wild-type HEK293T cells (**Figure 6**), which is consistent with a previous finding showing that the mRNA and protein levels of SGLT2 are minimal in the proximal tubular cell line (HK-2) in comparison to human kidney cells (30). Interestingly, we found that the mRNA levels of SGLT2 were increased in cells expressing mutant GLUT2 (**Figure 6**). Moreover, the accumulation of intracellular glucose was increased in the mutant cells (**Figure 7**). One possibility for the increased accumulation glucose in the mutant cells could be the enhanced expression of SGLT2 in these cells. To test this hypothesis, the glucose uptake assay was repeated in the presence of a clinically used SGLT2 inhibitor. As expected, the inhibitor attenuated glucose accumulation in both wild-type and mutant cells. However, the accumulation of glucose in the mutant cells remained higher than the wild-type cells even in the presence of the SGLT2 inhibitor, supporting our hypothesis that the GLUT2 mutant cells have a defect in glucose export.

In conclusion, our results support the notion that the last four transmembrane domains of GLUT2 (domains 9-12) are vital for glucose transport activity and suggest that disturbances in GLUT2 expression and/or function could contribute to the dysglycemia observed in FBS. It will be very important to explore if the intracellular accumulation of glucose that we observe in our gene edited HEK293T cells also results in the accumulation of glycogen, similar to the accumulation of glycogen in the kidneys observed in some FBS patients. In addition, future work will focus on using CRISPR-Cas gene editing to introduce FBS-associated GLUT2 mutations in other metabolically active human cells, including pancreatic beta and liver cell lines. Such studies will allow us to better understand the role of GLUT2 in FBS, and hopefully help in the development of better treatment options for FBS patients.

The limitation of our study is that we have only studied a single patient with a GLUT2 mutation. Similar studies should be undertaken in more patients with other GLUT2 mutations. Another limitation of our study is that we were unable to determine if the accumulation of glucose in our GLUT2 mutant HEK293T cells resulted in increased accumulation of glycogen in these cells. The most accurate method to monitor glycogen accumulation in non-liver cells is metabolic labeling using radioactive glucose. Unfortunately, the strict regulations controlling the use of radioactive materials in Qatar prevented us from performing these studies. Such studies should however be explored with collaborators outside Qatar in the future.

## Data Availability Statement

The datasets generated during and/or analysed during the current study are not publicly available to protect patient confidentiality but are available from the corresponding author on reasonable request.

## Ethics Statement

This study was approved by the Institutional Review Board for the Protection of Human Subjects, Sidra Medicine, Qatar and written informed consent was obtained for the study. Written informed consent to participate in this study was provided by the participants’ legal guardian/next of kin. Written informed consent was obtained from the minor(s)’ legal guardian/next of kin for the publication of any potentially identifiable images or data included in this article.

## Author Contributions

Writing the Manuscript: SS, MS, JE, and KH. CRISPR: SS, MS, IM, IH, MA, and KH. Patient recruitment and clinical data collection: SS, BH, and KH. Flow cytometry: SS, IP, SM, J-CG, and KH. Edu assay: SS, AB, SN, KB, and KH. WGS and Sanger sequencing: SS, IM, NS, and KH. Rest experimental work: SS, AA, JE, and KH. All authors read, edited, and approved the final manuscript for submission.

## Funding

This research was supported by the Qatar National Research Fund (QNRF-NPRP 10-6100017-AXX) awarded to KH. Work in the Ericsson laboratory is supported by the Qatar National Research Fund (NPRP13S-0127-200178).

## Conflict of Interest

The authors declare that the research was conducted in the absence of any commercial or financial relationships that could be construed as a potential conflict of interest.

## Publisher’s Note

All claims expressed in this article are solely those of the authors and do not necessarily represent those of their affiliated organizations, or those of the publisher, the editors and the reviewers. Any product that may be evaluated in this article, or claim that may be made by its manufacturer, is not guaranteed or endorsed by the publisher.
