# Supplementary figures and images for: Understanding the Mechanism of Dysglycemia in a Fanconi-Bickel Syndrome Patient
Source: Front Endocrinol (Lausanne). 2022 May 18;13:841788. doi: 10.3389/fendo.2022.841788 (PMC9159359; doi:10.3389/fendo.2022.841788)

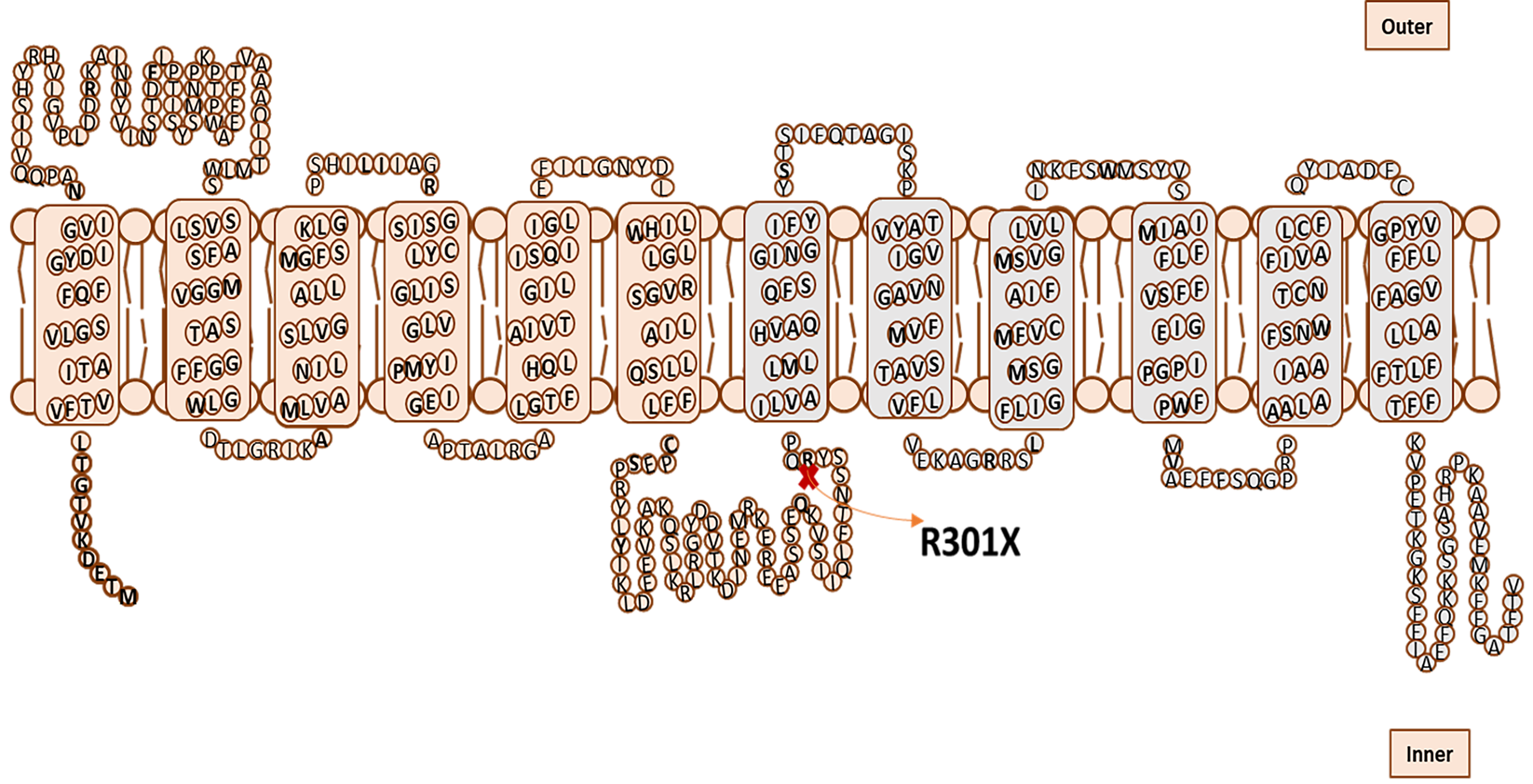

Supplement: Supplementary file 1 [file Image_1.tif]

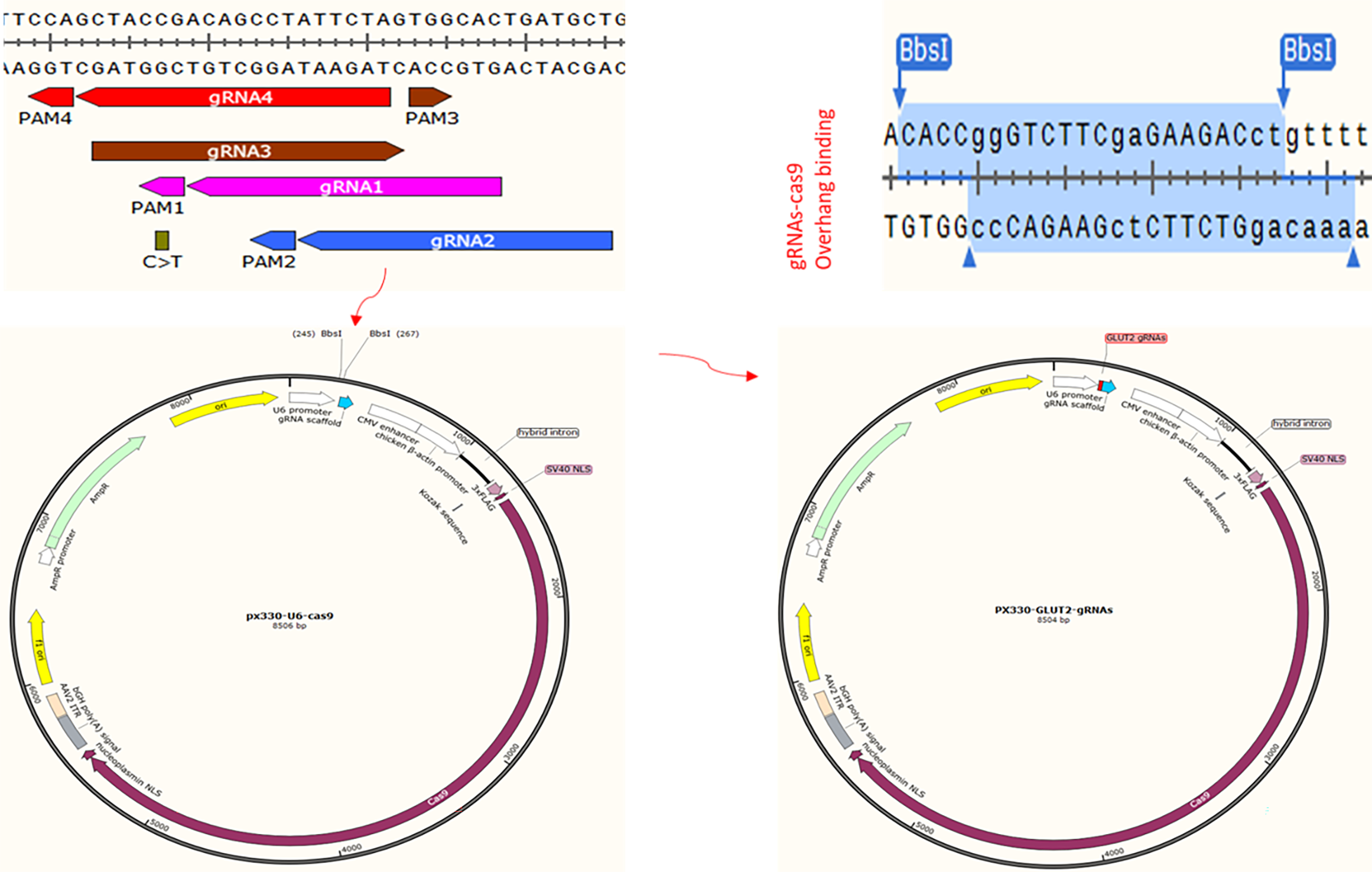

Supplement: Supplementary file 2 [file Image_2.tif]

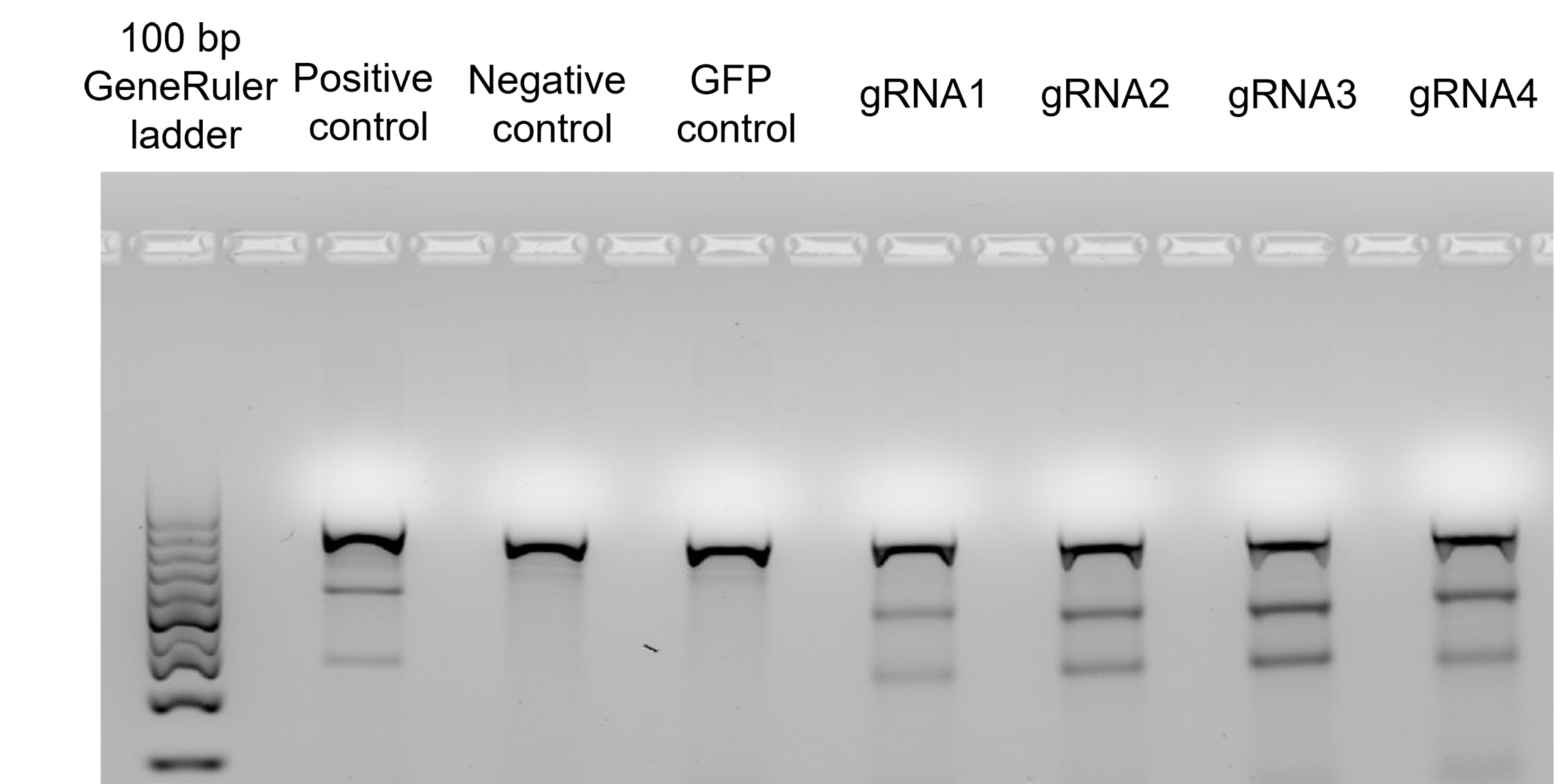

Supplement: Supplementary file 3 [file Image_3.tif]

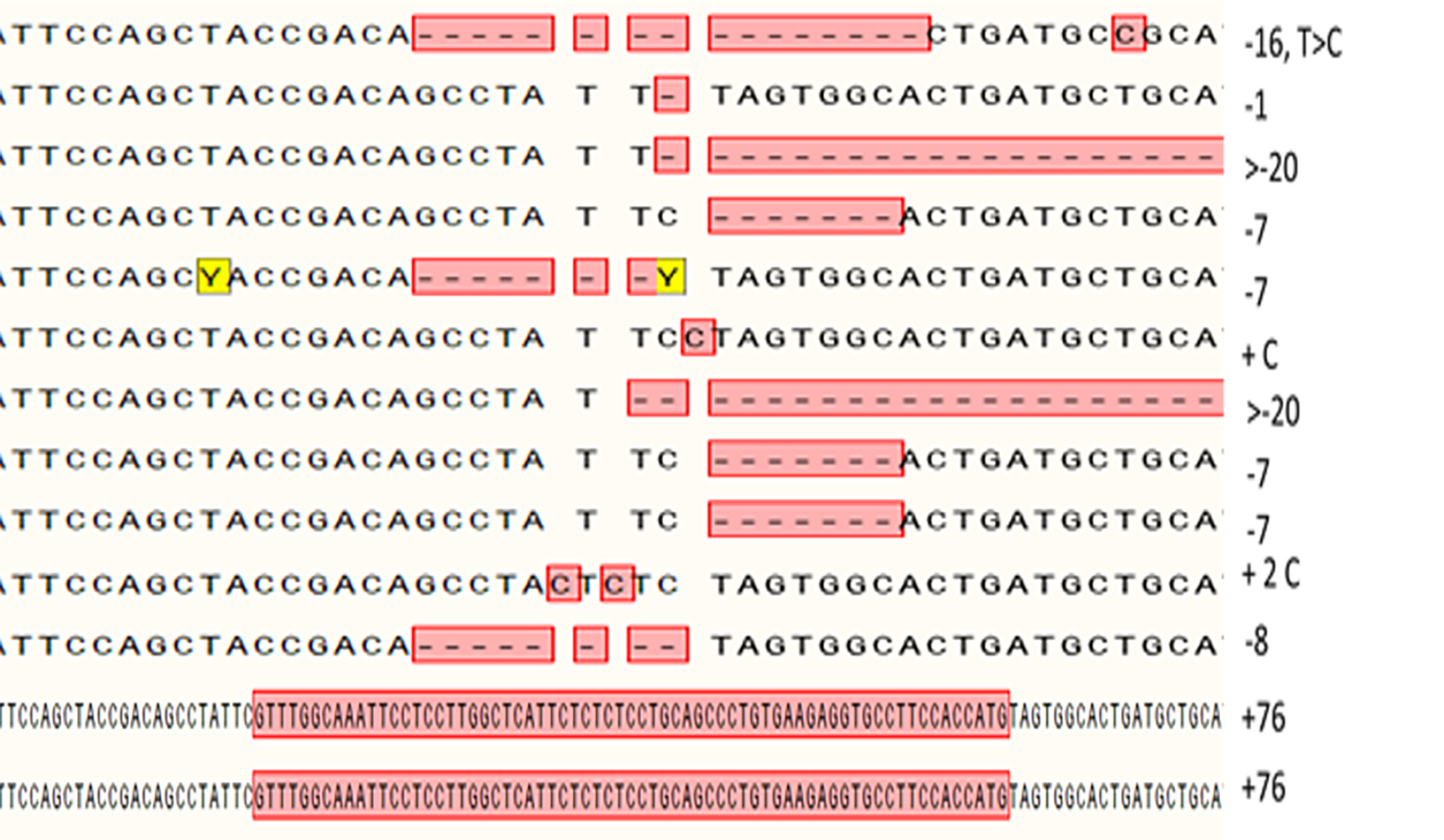

Supplement: Supplementary file 4 [file Image_4.tif]

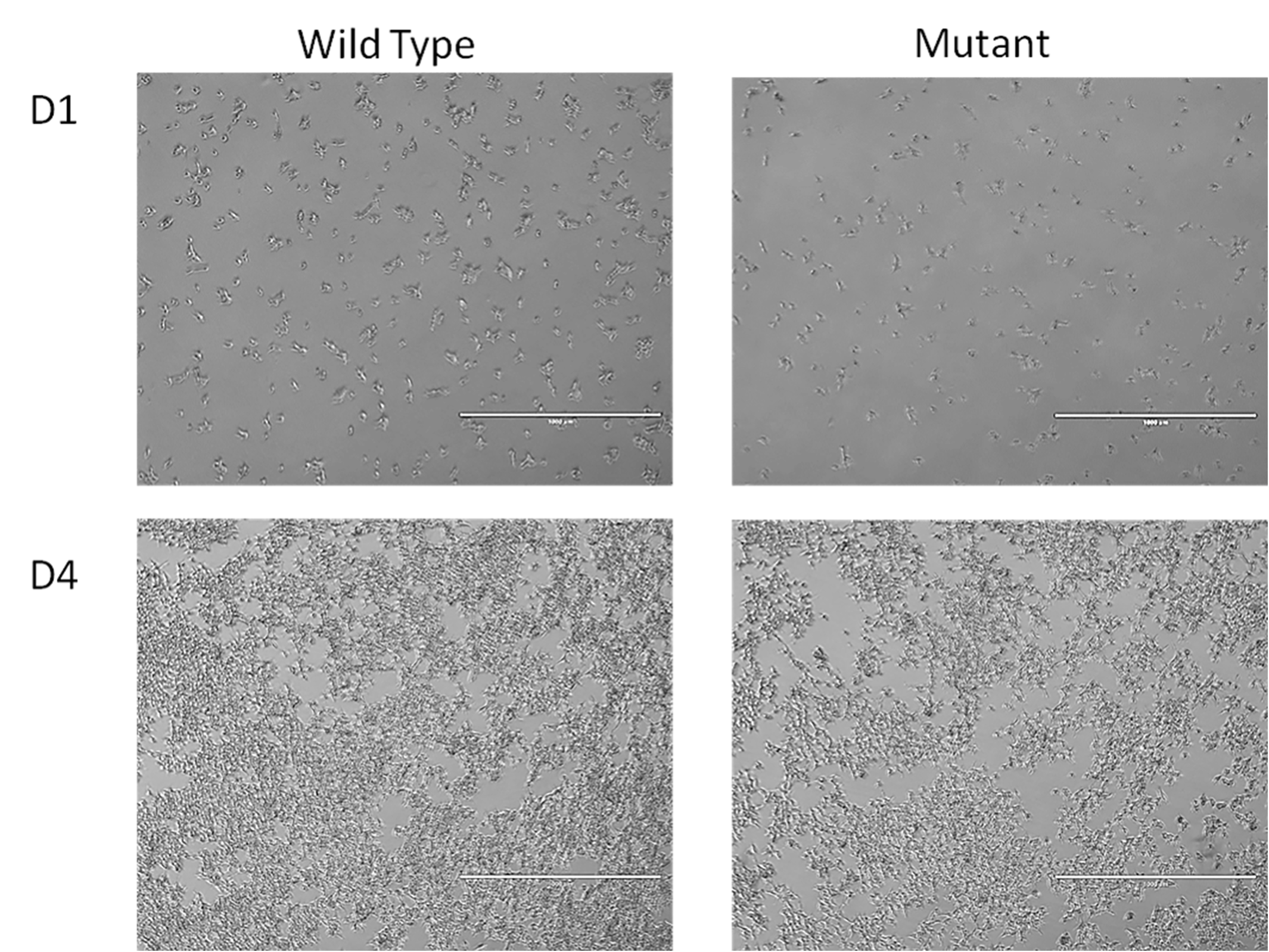

Supplement: Supplementary file 5 [file Image_5.tif]
