# Supplementary material for: Understanding the Mechanism of Dysglycemia in a Fanconi-Bickel Syndrome Patient
Source: Front Endocrinol (Lausanne). 2022 May 18;13:841788. doi: 10.3389/fendo.2022.841788 (PMC9159359; doi:10.3389/fendo.2022.841788)
Supplement: Supplementary file 6 [file Table_1.docx]

fable S1: List of primers used in experiments.

| Experiment | Forward Primer | Reverse Primer |
| --- | --- | --- |
| DNA Sanger Sequencing | | |
| db-bl-1164 | TGGCCTGAGTTGTTTCAACC | TTCCGGAAAATTGCTGAGCC |
| qRT PCR | | |
| GAPDH | CTGACTTCAACAGCGACACC | TTACTCCTTGGAGGCCATGT |
| GLUT1 | TGTCGTGTCGCTGTTTGTG | ATGGCCACGATGCTCAGATA |
| SGLT1 | ATGGCCTGGGTTCATCTTTG | GGCTGATCATTCCTGGCATC |
| SGLT2 | CATCTATGCCTCCGTCATCG | TGCTCCCAGGTATTTGTCGA |
| gRNAs | | |
| gRNA1 | caccGTGCCACTAGAATAGGCTGT | aaacACAGCCTATTCTAGTGGCAC |
| gRNA2 | caccGGCATCAGTGCCACTAGAAT | aaacATTCTAGTGGCACTGATGCC |
| gRNA3 | caccGTACCGACAGCCTATTCTAG | aaacCTAGAATAGGCTGTCGGTAC |
| gRNA4 | caccGAGAATAGGCTGTCGGTAGC | aaacGCTACCGACAGCCTATTCTC |
| CRISPR_Seq | | |
| gRNAU6 | GAGGGCCTATTTCCCATGATTCCT | CTAGAGCCATTTGTCTGCAGAATTGGC |
| Topocloning experiment | | |
| M13 | GTAAAACGACGGCCAG | CAGGAAACAGCTATGAC |
| Genomic GLUT2 | | |
| T7 | ACCCATGCTATTAAGTAATACAATGGGGCA | CCTAAATTCAATACTCAACTGTAGAAGCTCCTTGC |

Figure S1: Expected effect of patient’s mutation on GLUT2 topology (truncated protein). The last six loops of GLUT2 were truncated in the patient.

Figure S2: Schematic diagram for constructing the PX330-Cas9 plasmid by BbsI digestion. Four different gRNAs close to the patient mutation (c. 901C>T, R301X) were designed with complementary BbsI overhangs. Constructs containing the full gRNA (crRNA + tracrRNA scaffold) were validated by Sanger sequencing.

Figure S3: T7 assay to assess 4 different gRNAs activity. Editing activity is measured by the detection and cleavage of mismatches between pool of transfected HEK293T cells. gRNA3 was the most efficient gRNA to induce cuts at the GLUT2 DNA target site. Expected size of gRNAs fragments: (amplified sequence of interest): gRNA1, gRNA2, gRNA3, and gRNA4 respectively: (654); 259-395, 266-388, 247-407, and 252-402. Three independent experiments were run to confirm the result.

Figure S4: Sanger sequencing of Topo cloning colonies. 13 out of 22 colonies had a GLUT2 mutations caused by insertion and/or deletion of nucleotides due to NHEJ repair mediated by Cas9 DNA editing activity.

Figure S5: Morphological difference between WT and mutant HEK293T cells. Both cell types had the same morphology. However, WT cells grew faster than the mutant cells. Two independent experiments were run to confirm the result.
